# Phenolic Compounds from the Aerial Parts of *Blepharis linariifolia* Pers. and Their Free Radical Scavenging and Enzyme Inhibitory Activities

**DOI:** 10.3390/medicines6040113

**Published:** 2019-11-22

**Authors:** Amina Ibrahim Dirar, Mikiyo Wada, Takashi Watanabe, Hari Prasad Devkota

**Affiliations:** 1 Graduate School of Pharmaceutical Sciences, Kumamoto University, 5-1 Oe-honmachi, Chuo-ku, Kumamoto 862-0973, Japan; aminadirar2007@gmail.com (A.I.D.); wadayo@kumamoto-u.ac.jp (M.W.); wtakashi@kumamoto-u.ac.jp (T.W.); 2 Medicinal, Aromatic Plants and Traditional Medicine Research Institute (MAPTRI), National Center for Research, P.O. Box 2404, Mek Nimr Street, Khartoum 11111, Sudan; 3 Faculty of Clinical and Industrial Pharmacy, National University-Sudan, P.O. Box 3783, Al-Raki Area, Khartoum 11111, Sudan; 4 Program for Leading Graduate Schools, Health life Sciences: Interdisciplinary and Glocal Oriented (HIGO) Program, 5-1 Oe-honmachi, Chuo-ku, Kumamoto 862-0973, Japan

**Keywords:** *Blepharis linariifolia*, Sudan, phenolic compounds, free radical scavenging, α-glucosidase, pancreatic lipase, tyrosinase

## Abstract

**Background:***Blepharis linariifolia* Pers. (Family: Acanthaceae) is used in traditional medicines as a general tonic and for the treatment of various health problems in Sudan. The main aim of this study was to isolate and identify the major chemical constituents from the aerial parts of *B. linariifolia* and evaluate their bioactivities. **Methods:** The dried aerial parts of the plant were extracted successively with 100% acetone and 50% acetone, and thereafter the combined extract was subjected to repeated column chromatography to isolate the main components. Free radical scavenging activity was evaluated using the 1,1-diphenyl-2-picrylhydrazyl (DPPH) free radical method, and in vitro enzyme inhibitory activities against α-glucosidase, pancreatic lipase, and mushroom tyrosinase were evaluated. **Results:** From the detailed chemical analysis, verbascoside (**1**), vanillic acid (**2**), apigenin (**3**), and 6″-*O*-*p*-coumaroylprunin (**4**), were isolated and their structures were identified on the basis of their nuclear magnetic resonance (NMR) spectral data. Among the isolated compounds, verbascoside (**1**) showed the most potent free radical scavenging activity (IC50 = 22.03 ± 0.04 μM). Apigenin (**3**) and 6″-*O*-*p*-coumaroylprunin (**4**) showed promising inhibitory activities against all tested enzymes. Apigenin (**3**) showed the most potent inhibitory activity against α-glucosidase and tyrosinase (IC50 = 34.73 ± 1.78 μM and 23.14 ± 1.83 μM, respectively), whereas 6″-*O*-*p*-coumaroylprunin (**4**) showed potent inhibition for lipase (IC50 = 2.25 ± 0.17 μM). **Conclusions:** Four phenolic compounds were isolated and identified from *B. linariifolia* acetone extract, which are reported for the first time from this plant. All compounds showed good DPPH free radical scavenging activities, with verbascoside (**1**) being the most potent. Apigenin (**3**) was the most active as α-glucosidase and mushroom tyrosinase inhibitor, while 6″-*O*-*p*-coumaroylprunin (**4**) showed potent inhibitory activity for pancreatic lipase.

## 1. Introduction

*Blepharis linariifolia* Pers. (Family: Acanthaceae) is an annual herb widely distributed in many African countries, especially in areas from Mauritania to Sudan, and is also widely found across Arabia and India [1]. It is an edible herb consumed by human and ruminants [2]. Numerous ethnomedicinal uses have been reported for *B. linariifolia* among Africans, such as for healing skin burns and wounds, alleviating chest pain, and in treatments of urogenital infections. Further, the tisane of *B. linariifolia* is occasionally prescribed as a remedy for syphilis [1]. In Sudan, *B. linariifolia*, locally named “Begheil”, is distributed in Kordofan state in Western Sudan. The plant infusion is used as a general tonic, and for the treatment of stomach pain and bilharzia [3]. Moreover, the water extract of the fruits is used for urinary disorders and kidney stones [4,5]. The curative properties of *Blepharis* species paved the way for researchers to explore their therapeutic properties. Herein, several pharmacological properties for other plant species from the genus *Blepharis* have been reported, such as anti-inflammatory and anti-nociceptive properties [6], wound healing activities [7], and gastroprotective [8] effects of *B. maderaspatensis* (L.) B.Heyne ex Roth. Moreover, other studies reported antioxidant activity for *B. maderaspatensis* and *B. molluginifolia* Pers. [9]. Antimicrobial [10] and anticancer activities [11] were reported for *B. maderaspatensis.* Some studies on chemical analysis were reported for plant species from genus *Blepharis* and various phytochemical compounds were identified, such as stigmasterol tetracosanoate [12] and other flavonol glycosides, and blepharisides A and B [13] from *B. ciliaris* (L.) B.L.Burtt. As for the plant species *B. linariifolia*, a few studies have been conducted with respect to its therapeutic properties (for example antioxidant [14,15] and enzymatic inhibitory capacity [15,16]). Only one study has reported on a chemical analysis of the seeds of *B. linariifolia* [17].

Antioxidants are substances that scavenge free radicals and related reactive species. Free radicals amend vital body composition such as lipids and proteins, resulting in serious conditions such as inflammatory conditions, atherosclerosis, and cancerous diseases [18]. Therefore, dietary antioxidants are undertaken for enhancing health conditions for people under precarious sanitary conditions or to prevent serious and chronic illnesses such as neurodegenerative diseases, diabetes, and hyperlipidemia [19]. Diabetes mellitus (DM) is a metabolic disorder characterized by high blood glucose, which is likely to cause serious side effects such as retinopathy and cataracts. Thus, strategies were set for postponing the absorption of blood glucose via inhibition of carbohydrate-hydrolyzing enzymes such as α-glucosidase [20]. Obesity is another example of a metabolic disorder associated with several co-morbidities, with an increasing prevalence every year [21]. Excessive lipid accumulation could be one of the causative factors for type II diabetes, as the accumulation of lipid in the pancreas causes dysfunction of insulin-producing pancreatic β-cells [22]. Recently, inhibitors of pancreatic lipase have attracted interest due to their ability to delay lipid digestion, and hence could act as promising anti-obesity drugs [23]. Tyrosinase is a key enzyme in the process of melanin synthesis [24,25]. It has been isolated and identified from different sources [26,27]. Among them, mushroom tyrosinase, from *Agaricus bisporus*, is a major source and is widely used due to its high similarity and homology to human tyrosinase [28]. Despite the important protective role of melanin against UV-sunlight radiation [29], excess accumulation of this pigment results in harmful skin disorders and severe cases may cause skin cancer [30]. Thus, exploring new inhibitors with potential activities such as anti-diabetic, anti-lipidemic, or anti-melanogenic agents of natural origin have attracted widespread interest in recent years.

Previously, the effect of extraction solvents on the antioxidant and enzyme inhibitory activities was analyzed for six medicinal plants from Sudan [16]. Among the studied plants, 70% ethanol extract from the leaves of *Guiera senegalensis* J.F. Gmel. and acetone extract from the aerial parts of *B. linariifolia* showed promising activities. The detailed chemical analysis of *G. senegalensis* afforded eight phenolic compounds [31]. In continuation, the current study reports the isolation of phenolic compounds from the acetone extract of aerial parts of *B. linariifolia* and their antioxidant and α-glucosidase, pancreatic lipase, and mushroom tyrosinase inhibitory activities.

## 2. Materials and Methods

### 2.1. General Experimental Procedure

^1^H- and ^13^C-NMR spectra were measured on BRUKER AVANCE 600 NMR Spectrometer (Bruker, Billerica, MA, USA) (^1^H-NMR: 600 Hz and ^13^C-NMR: 150 Hz). Chemical shift values (δ_H_ and δ_C_) are given in ppm with reference to tetramethylsilane (TMS). Absorbance was recorded on ARVO MX 1420 Multilabel Counter Microplate Reader (Perkin-Elmer, Yokohama, Japan) at room temperature. Column chromatography (CC) was carried out with MCI gel CHP20P (75~150 μm, Mitsubishi Chemical Industries Co. Ltd., Tokyo, Japan), Sephadex LH-20 (Amersham Pharmacia Biotech, Tokyo, Japan), and silica gel 60 (0.040–0.063 mm, Merck KGaA, Darmstadt, Germany). Thin-layer chromatography (TLC) was performed on a pre-coated silica gel 60 F_254_ (Aluminum sheet, Merck KGaA, Darmstadt, Germany).

### 2.2. Plant Material

*B. linariifolia* aerial parts were collected from Kordofan state, West Sudan in February–April 2017. The plant was identified by Mr. Yahya Sulieman Mohamed, a taxonomist at the Medicinal, Aromatic Plants and Traditional Medicine Research Institute (MAPTRI), Khartoum, Sudan. The voucher specimen (voucher number: MPRTMI-H/O/41/81) was deposited at the Herbarium of MAPTRI, Khartoum, Sudan.

### 2.3. Chemicals

1,1-Diphenyl-2-picrylhydrazyl (DPPH) and the enzymes α-glucosidase (from *Saccharomyces cerevisiae*), porcine pancreatic lipase, and mushroom tyrosinase were purchased from Sigma Aldrich, Co. (Tokyo, Japan). 6-Hydroxy-2,5,7,8-tetramethylchroman-2-carboxylic acid (trolox) and acarbose were from Wako Pure Chemical Industries, Ltd. (Tokyo, Japan). Cetilistat was obtained from Combi-Blocks, San Diego, USA, and arbutin was purchased from Nacalai Tesque, Inc. (Kyoto, Japan). 2-Morpholinoethanesulfonic acid monohydrate (MES) was purchased from Dojindo Chemical Research (Kumamoto, Japan).

### 2.4. Extraction and Isolation

The shade-dried aerial parts of *B. linariifolia* (2 kg) were extracted with 100% acetone (30 L, two times) and then after with 50% acetone (16 L) at room temperature. The filtrated extracts were combined and evaporated under reduced pressure to give 184.0 g of extract (extract yield: 9.2% w/w). The extract was then subjected to MCI gel CHP20P column chromatography (CC) and eluted successively with water and 40%, 70%, and 100% MeOH to give eight fractions (Fr.1~Fr.8). Fraction 3 (4.90 g, water eluate) and fraction 4 (4.10 g, water eluate) were combined and subjected to Sephadex LH-20 CC and eluted with 50% MeOH to obtain six sub-fractions (SubFr. 3-1~SubFr. 3-6). Subfraction 3-4 (1.8 g, 50% MeOH eluate) was subjected to silica gel CC (CH_2_Cl_2_: MeOH: H_2_O = 9:1:0.1) and compound **1** (670 mg) was obtained. Compound **2** (10 mg) was isolated from sub-fraction 3-5-2 (0.05 g) by using silica gel CC (CH_2_Cl_2_: MeOH: H_2_O = 9:1:0.1). Fraction 6 (20.1 g, 70% MeOH eluate) was subjected to Sephadex LH-20 CC and eluted with MeOH to obtain four sub-fractions (SubFr. 6-1~SubFr. 6-4). Subfraction 6-3-2 (0.01 g) was subjected to silica gel CC (CH_2_Cl_2_: MeOH: H_2_O = 9:1:0.1) to obtain compound **3** (2.0 mg). Finally, compound **4** (38.7 mg) was obtained from sub-fraction 6-2-10 (0.29 g) from silica gel CC (CH_2_Cl_2_: MeOH: H_2_O = 9:1:0.1).

### 2.5. DPPH Free Radical Scavenging Activity

The antioxidant potential was determined using the 1,1- diphenyl-2-picrylhydrazyl (DPPH) radical scavenging method [32] with minor modifications. In a 96-well plate, 25 μL of compound solution of different concentrations were added, followed by the addition of 75 μL of 50% EtOH and 50 μL MES buffer (200 mM, pH = 6.0). Finally, 50 μL of freshly prepared DPPH solution in ethanol (800 μM) were added, and the assay plate was incubated for 20 min in the dark at room temperature. The antioxidant activity corresponding to scavenging of DPPH radicals was measured at 520 nm with UV spectrophotometer using the following formula: Radical scavenging activity (%) = 100 × (A − B)/A, where A is the control absorbance of DPPH radicals without samples and B is the absorbance after reacting with samples. Trolox was used as the positive control. From these data, curve was plotted and the inhibitory concentration (IC_50_) value was calculated.

### 2.6. In Vitro Enzyme Inhibitory Activities

#### 2.6.1. α-Glucosidase Inhibitory Activity

The α-glucosidase inhibitory activity was determined according to the method reported by Jabeen et al. [33] with slight modifications. In 96-well plate, 60 μL of 0.2 M phosphate buffer (pH = 6.8), 10 μL of the test sample (final concentration range of 100 to 1.56 μg/mL), and 10 μL of α-glucosidase solution (prepared in phosphate buffer to obtain 1 U/mL) were added. The mixture was incubated at 37 °C for 5 min and 20 μL of 4 mM *p*-nitrophenyl-α-_D_-glucopyranoside substrate were added. After 12 min of incubation at 37 °C, the released *p*-nitrophenol was measured at 405 nm using a spectrophotometer. The percentage inhibition of α-glucosidase activity was calculated using the equation: Inhibition (%) = (1 − (Aa − Ab)/(Ac − Ad)) × 100, in which Aa is the absorbance with test sample and enzyme, Ab is the absorbance with test samples but without enzyme, Ac is the absorbance with enzyme but without test sample, and Ad is the absorbance without test samples and enzymes. Acarbose was used as the positive control. From these data, curve was plotted and the inhibitory concentration (IC_50_) value was calculated.

#### 2.6.2. Pancreatic Lipase Inhibitory Activity

Evaluation of pancreatic lipase inhibitory activity was performed by measuring the release of 4-methylumbelliferone (4MUF) from the substrate 4-methylumbelliferyl oleate (4MUFO) as reported by Bitou et al. [34] with slight modifications. Briefly, 50 μL of the test sample and 50 μL of pancreatic lipase (100 μg/mL in phosphate buffer of 0.2 M, pH 7.4) were mixed and incubated at room temperature for 10 min. Afterward, 100 μL of the substrate 4MUFO (0.5 mM) was added. After 10 min, the fluorescence from the release of 4MUF was measured using a microplate reader with excitation and emission wavelengths of 355 and 460 nm, respectively. Cetilistat was used as the positive control. The percentage of pancreatic lipase inhibitory activity was calculated using the following equation: Inhibition (%) = (1 − (As/Ac) × 100), where As and Ac are the absorbance of sample and control, respectively. From these data, curve was plotted and the inhibitory concentration (IC_50_) value was calculated.

#### 2.6.3. Tyrosinase Inhibitory Activity

The tyrosinase inhibitory activity was evaluated using the method reported by Adhikari et al. [25], with slight modifications. In 96-well plate, 120 μL of phosphate buffer (50 mM, pH 6.8), 10 μL of test sample, and 50 μL of tyrosinase solution (100 U/mL in phosphate buffer) were added. The mixture was incubated at 25 °C for 10 min. Finally, 20 μL of the substrate _L_-tyrosine (2 mM) were added, and the absorbance was measured at 476 nm. The percentage of inhibition of tyrosinase activity was calculated using the equation: Inhibition (%) = (1 − (As/Ac)) × 100, where As represents the difference in the absorbance of the test sample between an incubation time of 10 and 2 min, and Ac represents the difference in the absorbance of the control sample between an incubation time of 10 and 2 min. Arbutin was used as the positive control. From these data, the curve was plotted and inhibitory concentration (IC_50_) value was calculated.

#### 2.6.4. Statistical Analysis

All the experiments were performed in triplicate. The data was analyzed using Microsoft Office Excel (2007) and results for DPPH free radical and in vitro enzyme inhibitory activities were expressed as IC_50_ value (μM).

## 3. Results and Discussion

**Figure 1:**
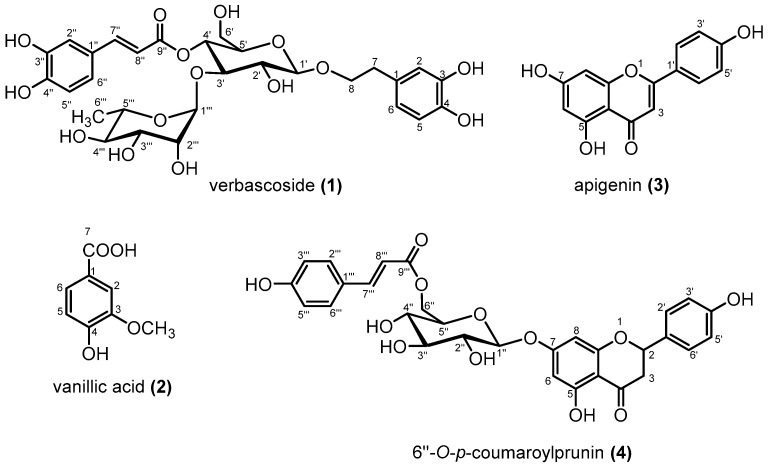
Chemical structures of compounds isolated from *Blepharis linariifolia*.

The combined 100% acetone and 50% acetone extract of aerial parts of *B. linariifolia* was subjected to various column chromatographic methods including MCI gel CHP20P, Sephadex LH-20, and silica gel, and four phenolic compounds were isolated. The chemical structures of the isolated compounds were elucidated as verbascoside (**1**) [35,36], vanillic acid (**2**) [37], apigenin (**3**) [38], and 6″-*O*-*p*-coumaroylprunin (**4**) [39] (Figure 1), on the basis of their NMR spectral data (Table 1, Table 2 and Table 3) and comparison to literature values. This is the first report for the isolation and identification of these phenolic compounds from *B. linariifolia*.

Verbascoside (also named as acteoside) is a phenylpropanoid glycoside distributed in many plants and widely isolated from *Verbascum* species [40], *Cistanche salsa* (C.A.Mey) Beck [35] and *Clerodendrun trichotomum* Thunb. [41]. In the current study, verbascoside (**1**) was isolated in high quantity (670 mg) in comparison to vanillic acid (**2**) (10.0 mg), apigenin (**3**) (2.0 mg), and 6″-*O*-*p*-coumaroylprunin (**4**) (38.7 mg), suggesting it is the main component from the acetone extract of *B. linariifolia*. Few other studies have also reported verbascoside from the genus *Blepharis*. For example, verbascoside was identified from the hydro-methanolic extract of *B. edulis* (Forssk.) Pers. [42] and the methanolic extract of *B. ciliaris* [43].

Vanillic acid (**2**) is distributed in numerous plant species [44]. Vanillic acid and its ester form were reported previously in *Blepharis* species, in which vanillic acid was identified from *B. edulis* [45], while methyl vanillate was identified from methanolic extract of *B. ciliaris* [43].

Apigenin (**3**) is a nutraceutical flavonoid distributed in many medicinal plants and functional foods [46]. Apigenin was reported in several species of *Blepharis*, where it was identified from the seeds of *B. sindica* Stocks ex T.Anderson [47]. Thereafter, one study reported apigenin in *B. ciliaris* [12], while the other study on *B. ciliaris* identified many glycosidic forms of apigenin [43,48]. 6″-*O*-*p*-Coumaroylprunin (**4**), also named prunin-6″-*O*-*p*-coumarate, has been previously identified from *B. sindica* [47] and *B. ciliaris* [43].

All of the isolated compounds were evaluated for their 1,1-diphenyl-2-picrylhydrazyl (DPPH) free radical scavenging and enzyme inhibitory activities, namely, α-glucosidase, porcine pancreatic lipase, and mushroom tyrosinase. IC_50_ (μM) values are given in Table 4. All compounds were active and showed diverse free radical scavenging activities and among them, verbascoside (**1**) was the most potent (IC_50_ = 22.03 ± 0.04 μM), and was found to be more potent than the positive control, trolox (IC_50_ = 39.07 ± 0.31 μM). Previous studies have also reported the antioxidant activity for verbascoside isolated from *B. edulis* [42] and *Halleria lucida* L. [49]. Verbascoside is a compound with a wide panel of pharmacological properties such as cytoprotective effect associated with alleviating and treatment of neurodegenerative diseases [50]. Interestingly, the neuroprotective effect was directly correlated to the free radical scavenging property along with other biological mechanisms [40,51]. Moreover, it was reported that medicinal plants with high concentration of verbascoside have pronounced anti-inflammatory and anti-microbial activities [40,52]. Herein verbascoside (**1**) was isolated as the major compound, and thus it is plausible to explain the traditional uses of *B. linariifolia* for inflammation and microbial infections [4] to the presence of this compound. As for the other compounds (**2**, **3**, and **4**) their antioxidant properties were confirmed in previous studies [53,54,55].

Regarding enzyme inhibitory activities, all compounds were evaluated for α-glucosidase, pancreatic lipase, and mushroom tyrosinase inhibitory activities (Table 4). Inhibition of α-glucosidase is widely acknowledged as a therapeutic indicator for controlling postprandial hyperglycemia and type II diabetes [56,57]. Thus, the α-glucosidase inhibitory activity was investigated for all compounds and it was found that apigenin (**3**) and 6″-*O*-*p*-coumaroylprunin (**4**) were more potent. Apigenin a renowned flavonoid found in many medicinal plants and functional foods [58]. The anti-diabetic property of apigenin was previously investigated [59], and recently the underlying mechanistic hypoglycemic effects were found to be attributed to inhibition of α-glucosidase along with other mechanisms [60,61]. Apigenin was also found to reversibly inhibit α-glucosidase in a noncompetitive manner [62]. As for 6″-*O*-*p*-coumaroylprunin (**4**), it also showed good α-glucosidase inhibitory activity. Literature revealed the paucity of pharmacological studies conducted so far, such as antinociceptive, antioxidant, anti-inflammatory, and antibacterial activities [55]. This is the first report for the α-glucosidase inhibitory activity of 6″-*O*-*p*-coumaroylprunin (**4**). As for the compounds verbascoside (**1**) and vanillic acid (**2**), neither showed inhibitory activities against α-glucosidase, although verbascoside was previously reported to possess α-glucosidase inhibitory activity [63]. Previously, vanillic acid was also reported as α-glucosidase inhibitor [64,65]. Different enzyme inhibitory activities are likely attributed to many factors such as initial sample concentration and concentration of substrate and incubation period with the enzyme [65].

The anti-lipidemic property for the compounds was assessed against porcine pancreatic lipase (PPL) enzyme and the anti-obesity drug cetilistat was used as a positive control. Herein, only compounds **3** and **4** were active in lipase inhibitory activity assay. These findings are strongly supported by previous studies exploring the anti-lipidemic property of apigenin (**3**) [66,67]. However, this is the first report of 6″-O-*p*-coumaroylprunin (**4**) as a potential lipase inhibitory agent. Although previous studies had reported the lipase inhibitory activities of verbascoside (**1**) [66] and vanillic acid (**2**) [68], they were inactive in this study.

As for tyrosinase inhibitory activity, all compounds, except for vanillic acid (**2**), showed good inhibitory activities preceding that of the standard arbutin. Apigenin (**3**) was the most active tyrosinase inhibitor (IC_50_ = 23.14 ± 1.83 μM), and the present finding also correlates to a previous report of its anti-tyrosinase potential [69]. Verbascoside (**1**) was also active as tyrosinase inhibitor, and this enzyme inhibitory activity was recently supported and molecularly explained for its effect on down-regulation of the tyrosinase enzyme [70]. Finally, 6″-*O*-*p*-coumaroylprunin (**4**) showed good anti-tyrosinase activity (IC_50_ = 136.12 ± 0.78 μM). Literature revealed limited pharmacological studies for **4**, reporting anti-inflammatory, antibacterial, and antinociceptive activities [55]. This is the first report of 6″-*O*-*p*-coumaroylprunin as potential tyrosinase inhibitor. As for vanillic acid (**2**), it did not show inhibitory activity against tyrosinase. A previous study reported a similar finding for the weak tyrosinase inhibitory activity of vanillic acid [71], while another study mentioned that vanillic acid did not show any inhibitory activity for mushroom tyrosinase [72].

It is worth to explore the correlation between the antioxidant and the foregoing enzyme inhibitory activities. Many studies stated that oxidative stress is a crucial determinant for type II diabetes complications, which had been explained through molecular mechanisms [73]. This could be clearly envisioned for the flavonoids apigenin (**3**) and 6″-*O*-*p*-coumaroylprunin (**4**). In contrast, verbascoside (**1**), which was the most potent antioxidant, did not show inhibitory activity for α-glucosidase in present study.

## 4. Conclusions

Four phenolic compounds, namely, verbascoside (**1**), vanillic acid (**2**), apigenin (**3**), and 6″-*O*-*p*-coumaroylprunin (**4**) were reported from the acetone extract of the aerial parts of *B. linariifolia* for the first time. All compounds showed free radical scavenging properties, with verbascoside (**1**) being the most potent. For enzyme inhibitory activities, both apigenin (**3**) and 6″-*O*-*p*-coumaroylprunin (**4**) were active as inhibitors for all enzymes. Apigenin (**3)** showed the most potent inhibition against α-glucosidase and tyrosinase, while 6″-*O*-*p*-coumaroylprunin (**4**) was mostly active as a lipase inhibitor. However, future studies should focus on their detailed mechanism of action using in vivo models for the development of potential therapeutic agents.

## Figures and Tables

**Table 1:** ^1^H and ^13^C-NMR spectroscopic data of verbascoside (**1**) in CD_3_OD.

Position	δ_C_	δ_H__,_ mult. (*J* in Hz)	Position	δ_C_	δ_H__,_ mult. (*J* in Hz)
1	131.5		1″	127.7	
2	116.5	6.69, d (2.0)	2″	114.7	7.05, d (2.0)
3	144.7		3″	149.8	
4	146.1		4″	146.8	
5	117.1	6.67, d (8.0)	5″	116.3	6.77, d (8.2)
6	121.3	6.56, dd (2.0, 8.0)	6″	123.2	6.95, dd (2.0, 8.3)
7	36.6	2.79, m	7″	148.0	7.59, d (15.8)
8	72.4	4.05, dd (8.7, 16.3) 3.72, dd (8.2,16.3)	8″	115.3	6.27, d (15.8)
1′	104.2	4.37, d (8.0)	9″	168.3	
2′	76.2	3.39, dd (8.0, 9.1)	1′′′	103.1	5.18, d (1.6)
3′	81.7	3.81, t (9.2)	2′′′	72.3	3.91, m
4′	70.4	4.91, t (9.5)	3′′′	72.1	3.55, m
5′	76.1	3.53, m	4′′′	73.8	3.35, m
6′	62.4	3.61, m	5′′′	70.6	3.52, m
			6′′′	18.5	1.09, d (6.2)

Abbreviations—δ_C_: chemical shift (in ppm) for ^13^C-NMR, δ_H_: chemical shift (in ppm) for ^1^H-NMR, mult.: multiplicity, *J* in Hz: coupling constant in Hz, d: doublet, dd: double doublet, m: multiple, t: triplet.

**Table 2:** ^1^H and ^13^C-NMR spectroscopic data of compounds **2** and **3** in CD_3_OD.

Position	Vanillic Acid (2)		Apigenin (3)	
	δ_C_	δ_H__,_ mult. (*J* in Hz)	δ_C_	δ_H__,_ mult. (*J* in Hz)
1	127.9		-	
2	114.0	7.51, brs	166.3	
3	149.6		103.9	6.59, s
4	147.8		183.9	
5	115.6	6.87, d (8.2)	163.2	
6	124.4	7.45, brd (8.2)	100.1	6.21, d (2.1)
7	174.5		166.0	
8			95.0	6.45, d (2.1)
9			159.4	
10			105.3	
1′			123.3	
2′ and 6′			129.4	7.85, d (8.8)
3′ and 5′			117.0	6.93, d (8.8)
4′			162.7	
OCH_3_	56.7	3.86, s		

Abbreviations—δ_C:_ chemical shift (in ppm) for ^13^C-NMR, δ_H_: chemical shift (in ppm) for ^1^H-NMR, mult.: multiplicity, *J* in Hz: coupling constant in Hz, brs: broad singlet, d: doublet, brd: broad doublet, s: singlet.

**Table 3:** ^1^H and ^13^C-NMR spectroscopic data of compound **4** in CDCl_3_.

Position	δ_C_	δ_H__,_ mult. (*J* in Hz)	Position	δ_C_	δ_H__,_ mult. (*J* in Hz)
2	79.7	5.29, dd (3.0, 12.6)	1″	100.5	4.98, d (7.4)
3	43.6	2.72–3.10, m	2″	73.8	3.38–4.58
4	197.6		3″	77.2	3.38–4.58
5	164.3		4″	71.0	3.38–4.58
6	96.8	6.18, d (2.2)	5″	75.1	3.38–4.58
7	166.2		6″	64.2	4.32, m
8	96.7	6.25, d (2.2)	1′′′	126.6	
9	163.6		2′′′ and 6′′′	130.7	7.38, d (8.7)
10	104.6		3′′′ and 5′′′	116.4	6.81, d (8.7)
1′	129.9		4′′′	160.4	
2′ and 6′	128.4	7.26, d (8.5)	7′′′	146.5	7.60, d (15.9)
3′ and 5′	116.1	6.84, d (8.5)	8′′′	114.4	6.32 d (15.9)
4	158.2		9′′′	168.7	

Abbreviations—δ_C:_ chemical shift (in ppm) for ^13^C-NMR, δ_H_: chemical shift (in ppm) for ^1^H-NMR, mult.: multiplicity, *J* in Hz: coupling constant in Hz, d: doublet, dd: double doublet, m: multiple.

**Table 4:** IC_50_ (μM) values of compounds isolated from *B. linariifolia* for their bioactivities.

Compounds	DPPH Free Radical Scavenging Activity	Enzyme Inhibitory Activities		
		α-Glucosidase	Lipase	Tyrosinase
Verbascoside (**1**)	22.03 ± 0.04	NA ^1^	NA ^1^	97.44 ± 2.48
Vanillic acid (**2**)	217.02 ± 3.01	NA ^1^	NA ^1^	NA ^1^
Apigenin (**3**)	386.29 ± 3.42	34.73 ± 1.78	12.46 ± 2.04	23.14 ± 1.83
6″-*O*-*p*-Coumaroylprunin (**4**)	186.24 ± 1.19	46.30 ± 2.92	2.25 ± 0.17	136.12 ± 0.78
Positive controls	39.07 ± 0.31 (Trolox)	565.56 ± 1.54 (Acarbose)	18.40 ± 1.71 (Cetilistat)	435.98 ± 3.71 (Arbutin)

^1^ not active.
